# Similarity matching of medical question based on Siamese network

**DOI:** 10.1186/s12911-023-02161-z

**Published:** 2023-04-06

**Authors:** Qing Li, Song He

**Affiliations:** 1 Medical College, Guizhou University, Guiyang, 550025 Guizhou Province China; 2 Information Department, The People’s Hospital of Guizhou Province, Guiyang, 550002 Guizhou China

**Keywords:** Siamese network, BiGRU, Ethnic medicine, Similarity matching, Medical questions

## Abstract

**Background:**

With the rapid development of the medical industry and the gradual increase in people’s awareness of their health, the use of the Internet for medical question and answer, to obtain more accurate medical answers. It is necessary to first calculate the similarity of the questions asked by users, which further matches professional medical answers. Improving the efficiency of online medical question and answer sessions will not only reduce the burden on doctors, but also enhance the patient’s experience of online medical diagnosis.

**Method:**

This paper focuses on building a bidirectional gated recurrent unit(BiGRU) deep learning model based on Siamese network for medical interrogative similarity matching, using Word2Vec word embedding tool for word vector processing of ethnic-medical corpus, and introducing an attention mechanism and convolutional neural network. Bidirectional gated recurrent unit extracts contextual semantic information and long-distance dependency features of interrogative sentences; Similar ethnic medicine interrogatives vary in length and structure, and the key information in the interrogative is crucial to similarity identification. By introducing an attention mechanism higher weight can be given to the keywords in the question, further improving the recognition of similar words in the question. Convolutional neural network takes into account the local information of interrogative sentences and can capture local position invariance, allowing feature extraction for words of different granularity through convolutional operations; By comparing the Euclidean distance, cosine distance and Manhattan distance to calculate the spatial distance of medical interrogatives, the Manhattan distance produced the best similarity result.

**Result:**

Based on the ethnic medical question dataset constructed in this paper, the accuracy and F1-score reached 97.24% and 97.98%, which is a significant improvement compared to several other models.

**Conclusion:**

By comparing with other models, the model proposed in this paper has better performance and achieve accurate matching of similar semantic question data of ethnic medicine.

## Background

In an age of medical data explosion, people are now increasingly concerned about their health status and are accessing the internet for information on strategies to address their ailments, with the emergence of various medical platforms to offer answers to their questions. Nevertheless, many online consultation systems fail to provide accurate feedback to the user, mainly because they do not match the exact type of question, resulting in a non-responsive answer. Question-sentence similarity matching is at the core of the question and answer system, and only when similar question sentences are matched will the appropriate answer be delivered to the user [1]. In the domain of natural language processing, semantic similarity is a relatively successful approach [2]. Text similarity is mainly used to measure the degree of semantic similarity between two sentences, eliminating repetitive and nonsensical information. The semantic similarity between sentences is based on their meanings to evaluate the degree of association between them [3].

Sentence similarity computation depends strongly on text comprehension and feature information extraction [4] and has been widely applied in fields such as summarizing [5], text classification [6, 7], search engines [8], and question and answer systems [9, 10]. Semantic similarity computation models in NLP-based domains are mainly divided into traditional text similarity [11, 12] computation and neural network similarity computation models. Traditional text similarity algorithms focus on TF-IDF [13], N-gram [14], Simhash [15], Jaccard [16] similarity, etc. These algorithms are relatively simple and convenient to implement, but they ignore the semantic information of the sentences, with comparatively inferior processing and generalization capabilities for text similarity semantics, which fail to extract text features correctly and may also give rise to a series of problems such as feature vector sparsity and dimensional explosion. On the contrary, the deep learning-based text similarity model mainly tackles the issue that traditional text similarity algorithms fail to adequately capture the contextual relationship of text semantics and extract critical feature information between texts.

Domestic and foreign scholars use CNN [17], RNN [18], LSTM [19], and many other models to study text similarity. These models are free from complex feature engineering and have well migration and adaptability. The DSSM (Deep Structured Semantic Models) model presented by Huang [20] et al. was the pioneer in applying the Siamese network [21] as a fundamental framework for text semantic similarity computation and attained relatively excellent results, but ignored the textual sequential and contextual information. Shen [22] et al. built on Huang by adding CNN (Convolutional Neural Networks), with convolutional and pooling layers to their model, preserving the word sequence information of the text, but disregarding the longer distance text features and only extracting local position-invariant feature information of the sentences [23]. Palangi [24] et al. incorporated Long-Short-Term Memory in the Siamese network architecture, which enables the extraction of long sequences of textual information and obtains global feature information, addressing the problem that RNN is unable to learn the information dependency of text over long distances. Mueller [25] et al. proposed a Siamese-LSTM network model to compute sentence semantic similarity, which firstly vectorizes the data, encodes different sentences into fixed-size features via two weight-sharing LSTM networks, and then uses the Manhattan distance [26] to measure the spatial similarity between the two sentences after obtaining the feature representation of the two sentences. Neculoiu [27] et al. also relied on Siamese networks, using a BiLSTM network based on character-level embedding to process sentence pairs, extracting semantic features of sentences that can learn semantic differences and semantic invariance among different words, then finally perform calculations with the cosine similarity to obtain the smallest distance in the space between vector embedding of similar sentence pairs and the largest distance between dissimilar pairs. In this paper, we substitute the BiLSTM with BiGRU based on the Siamese-BiLSTM model. For the LSTM with equivalent performance, the GRU [28] model has one less gating unit, fewer parameters, simpler structure, fewer training samples, faster, more easily implemented, stronger convergence, etc. At the same time, the attention mechanism and CNN model are inserted, and the dropout layer [29] is added to the structure of BiGRU, which is mainly to prevent the disappearance of the long-term memory built in the GRU’s unit and to avoid overfitting of the model. It can perform probabilistic deactivation of the input and recurrent connections of the bidirectional GRU neurons to further improve the performance of the model. CNN can extract local position invariant features of interrogative sentences, and the attention mechanism is to assign higher weights to relevant important semantic word vector features to further improve the recognition ability of interrogative sentences. In this paper, we constructed datasets of ethnic medical interrogative sentences to achieve accurate matching of ethnic medical similar semantic interrogative data, and meanwhile, we also use the Novel Coronary Pneumonia 2019 medical interrogative sentences public datasets to verify the effectiveness of the model proposed in this paper. In this study, ethnic medicine was chosen mainly because the project content of this study is centered on the techniques, methods, prescriptions, and research on the prevention and treatment of common diseases by ethnic minority medicine. Therefore, collecting ethnic medicine data is very important in the experimental process. Secondly, ethnic medicine is an essential material cultural heritage of China, and it is the medical experience acquired by ethnic minority people through years of practice. Strengthening the collation and protection of ethnic medicine can not only contribute to the development of local ethnic medicine but also promote the inheritance and development of ethnic medicine.

This paper contains the following sections. The first section deals with ethnic medicine data, the second with the model structure and data training based on the Siamese network, and the third with the parameter settings for the experiments and the results of the experimental analysis of the comparative models. The last part is the conclusion.

## Method

### Data acquisition and processing

Ethnic medicine is the traditional medicine of China’s ethnic minorities. However, the prescriptions of ethnic medicine have great variability among ethnic groups. To preserve the heritage and development of ethnic medicine, an ethnic medicine question and answer system is constructed, which can facilitate the full excavation and collation of ethnic medicine data and provide a broader learning channel for ethnic medicine medical practitioners and researchers. The most crucial aspect of the Ethnic Medicine Q&A system is question similarity matching. The major types of data include unstructured data, semi-structured data and structured data. Structured data is well organized and with well-defined relationships, but such accurate data is rare. Therefore, ethnic medicine data is available predominantly through semi-structured and unstructured methods. The semi-structured data was acquired through web crawling techniques to obtain ethnic medicinal question and answer data, researching the main functions of Chinese medicine web pages and ethnic medicinal web pages, discovering that the data on ethnic medicine was very sparse, and storing the relevant data as an ethnic medicinal corpus, carrying out data cleaning based on the crawled ethnic medicinal prescriptions and online question and answer data, removing useless and duplicate data, modifying mistaken data, adding missing data, making correct conversions for Chinese and English symbols, cleaning up deactivated words and invalid fields in the text, and constructing about 1000 ethnic medicine question and answer data. The unstructured data was mainly collected from ethnic medicine books, medical records of the Guizhou Provincial Hospital of Traditional Chinese Medicine, and doctors’ answers to patients, through the gathering and collation of these data, more than 1,500 questions and answers data were generated, and the data obtained from these two sources were combined to build a total of over 2,500 ethnic medicine questions and answers data (Q–A data).

Expanding on this foundation with pairs of interrogative sentences, the Q-Q data set contains question 1 (Q1), question 2 (Q2) and question labels. If the semantics of the text represented by question 1 and question 2 is similar, the label is 1, which is a positive sample data otherwise it is 0, which is a negative sample data. Based on more than 2,500 question sentences to be extended, for the set of question sentences Q_n_ = {q_n1_,q_n2_,q_n3_,…,q_nn_}, one question sentence at a time is selected for small language translation, and each question will approximately take any five different small languages for translation, and then translated back to Chinese, the similar question sentence pair Q_s_ can be obtained as a positive sample, and for negative samples, they are combined with other different semantic question sentences in the set Q_n_ to gain a non-similar question sentence pair Q_i_. A total of 22,655 ethnic medicine question pairs were obtained, and Q_s_ and Q_i_ were randomly disrupted during training, with a positive and negative sample of approximately 1:1. The question pair data are shown in Table 1.

**Table 1:** Example of ethnic medicine question pair data

Q1	Q2	Question label	Data type
(How to use Cordyceps Sinensis in chicken soup, for how long and in what quantity? What are the main effects and can older people take it?)	(How to use the valuable Tibetan herb Cordyceps Sinensis in chicken soup, how long and how much is better? What are the main effects and can the elderly take it?)	1	Positive sample
(What are the precautions to take when taking a Yao herbal bath, and which herbs are mainly used to have an obvious effect on beauty and whitening, and can I soak regularly?)	(Is Yi medicine Hungry Seeking EqiuQi tablets effective in treating diarrhea-type chronic enteritis, how many courses of treatment do I need to take and will it come back?)	0	Negative sample

### Siamese BiGRU attention CNN model

**Fig. 1:**
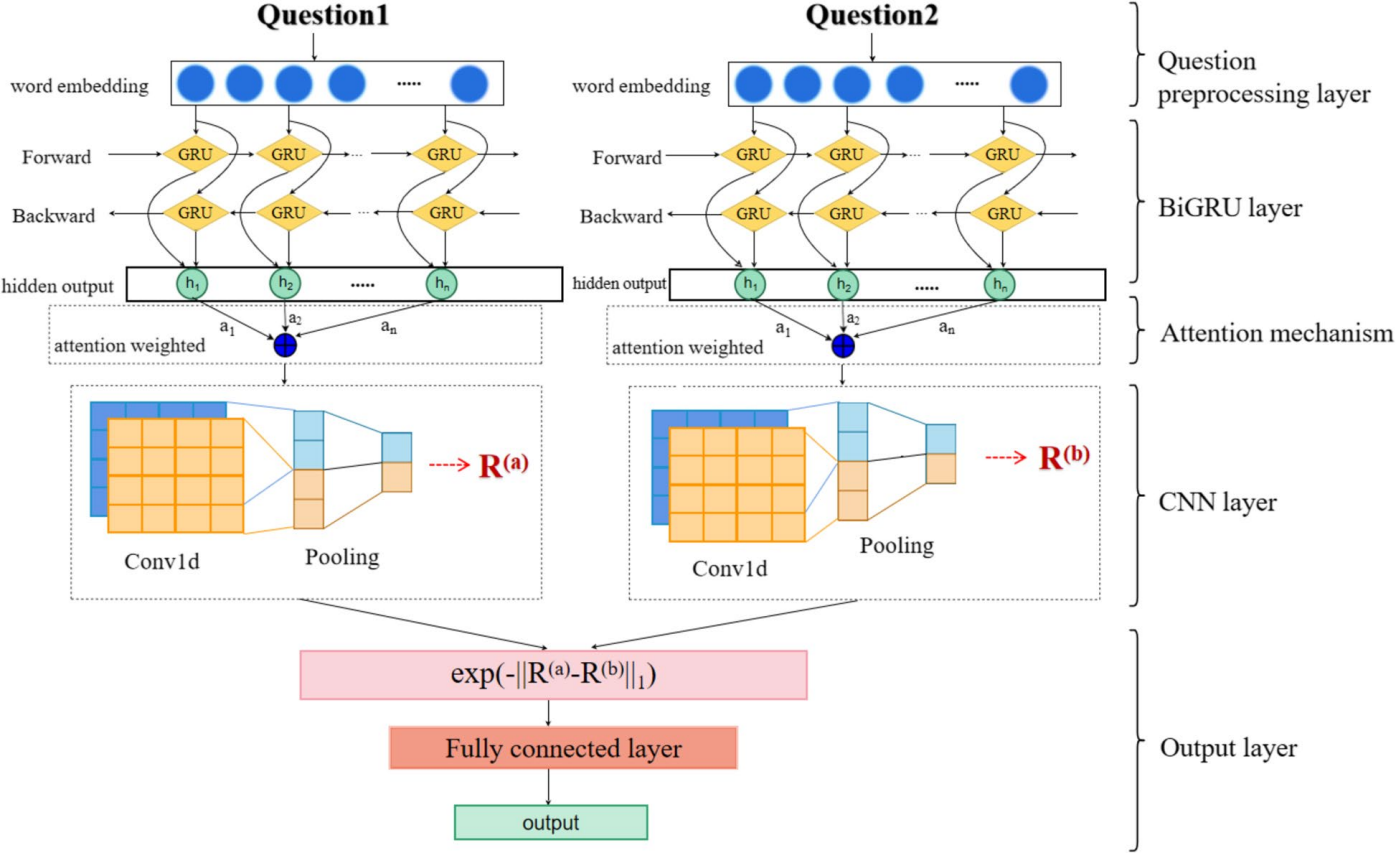
Structure of Siamese-BiGRU-Attention-CNN model

In this paper, the Siamese-BiLSTM network model is improved by replacing the BiLSTM with a BiGRU model, and adding an attention mechanism and a convolutional neural network, as shown in Fig. 1. The network model that is proposed in this paper has five main layers: the interrogative preprocessing layer, the BiGRU layer, the Attention [30] layer, the CNN layer, and the output layer. In comparison to long and short-term memory models, GRU is simpler in structure, more achievable and takes less time to train. BiGRU receives data from the forward and reverse directions and extracts contextual information and semantic features from the interrogative sentences. The attention mechanism can enhance the semantic information of the keywords, which contributes to the CNN model to capture the local position invariant features of the interrogative sentences, then finally the spatial similarity between two interrogative sentences is calculated by Manhattan distance.

#### Siamese network

**Fig. 2:**
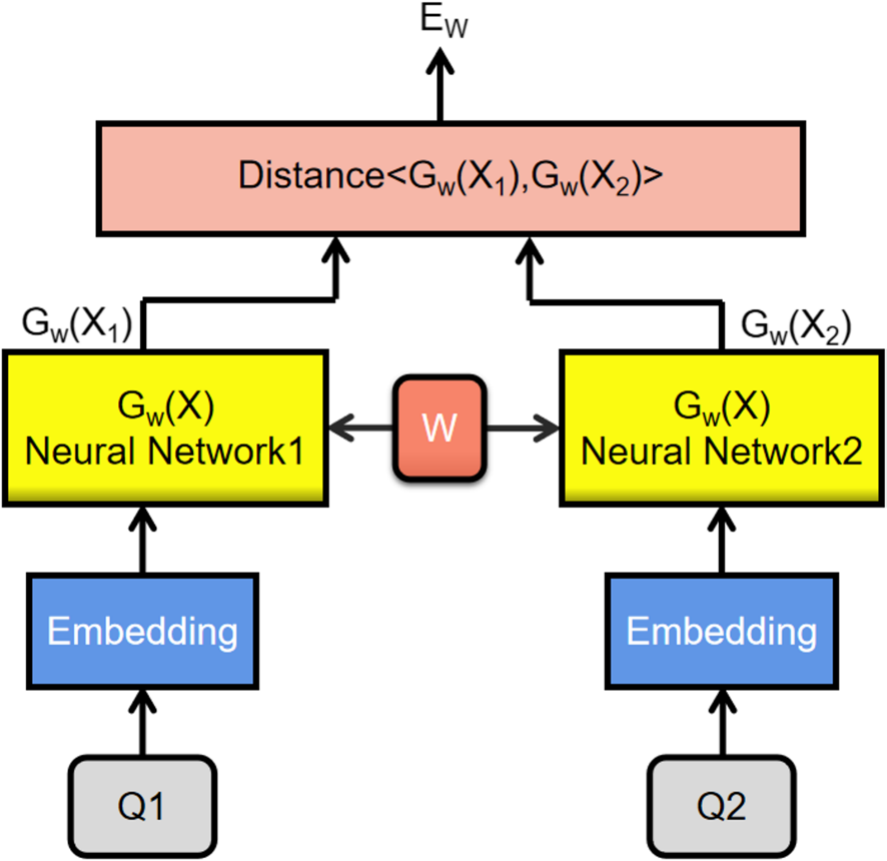
Siamese network structure

The Siamese network is a conjoined neural network with two identical structures and shared weights [31], originally applied in the field of image processing. In the field of natural language processing, the main purpose is to measure the semantic similarity of two input texts. The neural networks in Siamese can be RNN, CNN, LSTM and other models to train the datasets and get the feature vectors, the semantically similar question vectors will be mapped to the similar vector space, and the similarity can be calculated by the vector distance formula. The overall structure of the conjoined neural network is shown in Fig. 2.

#### Pre-processing of interrogative word embedding

Deep learning networks cannot receive text data directly and need to convert the interrogative sentences into numerical vectors first. In order to retain the comprehensive and complete semantic information of the interrogative sentences, the interrogative datasets are firstly pre-processed to remove the redundant information and data noise of the text, which is different from English utterances in that there are spaces between words and the Chinese utterances are all continuous, and therefore it is necessary to do the word separation process first. Most of the current word splitting tools such as LTP from Harbin Institute of Technology [32] and the Jieba tool [33] in Python have a high accuracy rate. In this paper, we utilize the Jieba word splitting tool to split ethnic medicine interrogative sentences and train the ethnic medicine corpus to be used as a splitting dictionary at the same time, so that the ethnic medicine professional vocabulary can be properly split and the recognition ability of professional domain vocabulary can be further strengthened. The question is divided before and after as shown in Table 2, using spaces as separators.

**Table 2:** Examples of pre-processing results for ethnic medical interrogatives

Pre-processing questions	Post-processing questions
(How do Dragon Date Capsules from Chinese Ethnic Medicine work, how do they work and what are the precautions to take while taking them?) 中国民族医药的龙枣胶囊功效如何, 怎么使用, 服用期间有什么注意事项?	(How do Dragon Date Capsules from Chinese Ethnic Medicine work, how do they work and what are the precautions to take while taking them ?) 中国 民族 医药 的 龙枣 胶囊 功效 如何, 怎么 使用, 服用 期间 有 什么 注意事项 ?
(Where can I buy Daiyao Xie Sha (Bai Xie Capsules), an ethnic medicine, other than Xishuangbanna, and how much does it cost?) 请问, 傣药雅解沙(百解胶囊)这类民族医药在哪里有卖,除了西双版纳之外, 代购大概需要多少钱, 贵不贵?	(Where can I buy Daiyao Xie Sha ( Bai Xie Capsules), an ethnic medicine, other than Xishuangbanna, and how much does it cost ?) 请问, 傣药 雅解沙 ( 百解 胶囊) 这类 民族 医药 在 哪里 有 卖, 除了 西双版纳 之外, 代购 大概 需要 多少 钱, 贵不贵 ?
(Which of the four major ethnic groups is the ancient formula for hair care that has been handed down in China for thousands of years, and which of the four major ethnic groups of medicine is used by Shangkang Yuan to prevent hair loss?) 中国千年传承的千年育发古方, 尚康源防脱育发采用的四大民族医药是哪个名族?	(Which of the four major ethnic groups is the ancient formula for hair care that has been handed down in China for thousands of years, and which of the four major ethnic groups of medicine is used by Shangkang Yuan to prevent hair loss ?) 中国 千年 传承 的 千年 育发 古方, 尚康源 防脱育发 采用 的 四大 民族 医药 是 哪个 名族 ?

The popular word embedding methods are Word2vec [34], ELMo [35], Glove [36], BERT [37], etc. Word2vec is an open-source word embedding tool proposed by Google as a word vector pre-training model, which is divided into two major types: CBOW(continuous bag-of-words model) model and Skip-gram(continuous skip-gram model) model [38]. CBOW is predicting target words based on context while Skip-gram is predicting context according to target words In this paper, the Skip-gram model is used for the vectorized representation of ethnic medicine interrogatives as input to the BiGRU neural network layer. Skip-gram makes a vector representation using the target word and the surrounding context words in a pre-defined sliding window. By maximizing the hits of the target and context word vectors and going through the sigmoid function, the gradient is calculated for reverse iterate, updating the learning weights and continuously optimizing the model. Mapping semantically similar word vectors to similar locations in space solve the vector sparsity problem and gains access to low-dimensional vector data.

#### Bidirectional gated recurrent unit

A Gated Recurrent unit neural network (GRU) is a variant model of LSTM that addresses the problems of gradient decay and explosion in recurrent neural networks while capturing long-distance dependency in text sequences. With relatively comparable performance, GRU simplifies the internal structural units, cuts parameters, converges faster, and is more readily implemented than LSTM.

**Fig. 3:**
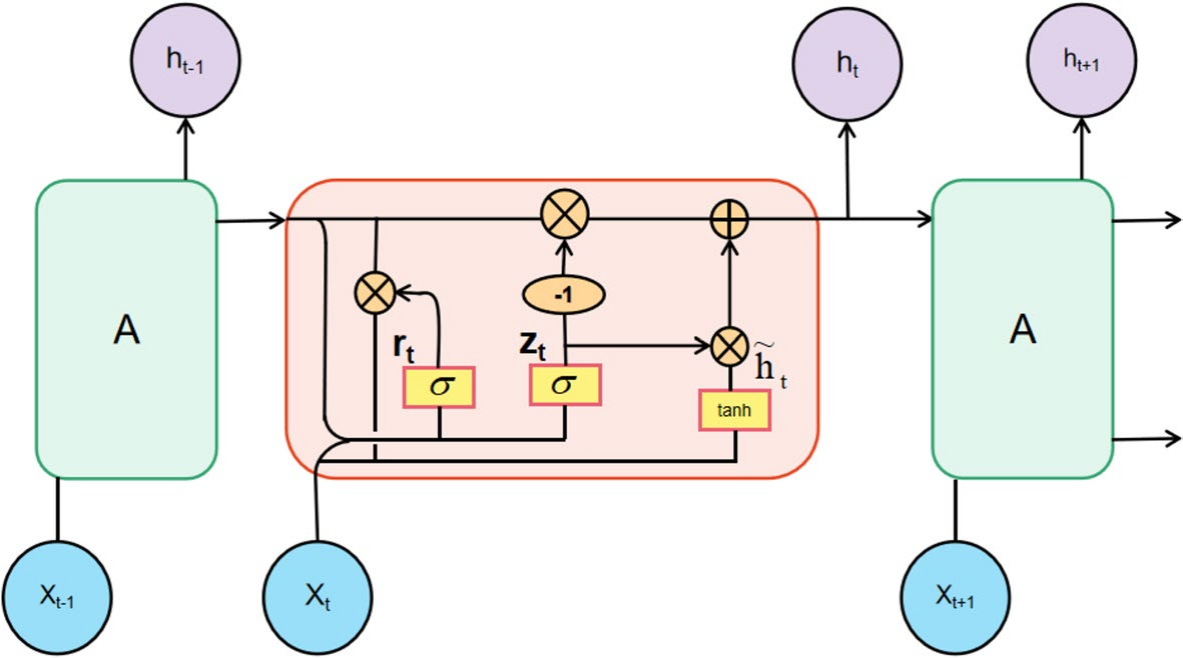
Structure of the GRU unit

The GRU model has two main control gates, the update gate (${z}_{t}$) and the reset gate (${r}_{t}$), which combine the forgetting gate and the input gate in the LSTM into an update gate. The update gate facilitates the capture of long-term dependencies in the time series, with the reset gate being beneficial to the capture of short-term dependencies in the time series. A diagram of the GRU unit structure is displayed in Fig. 3.

In the following equation: ${x}_{t}$ is the input word vector at moment t, ${h}_{t}^{^{\prime}}$ is the candidate state at the current moment, ${h}_{t}$ is the hidden state at the current moment, ${h}_{t-1}$ is the hidden state at the previous moment, ${r}_{t}$ is the reset gate, merging the new input with the original information, ${z}_{t}$ is the update gate, ${W}_{r}$ and ${W}_{z}$ are the weight matrices, $\sigma$ means sigmoid activation function.

The first one is the reset gate, which is controlling the degree of dependency between the candidate state ${h}_{t}^{^{\prime}}$ at the current moment and the hidden state at ${h}_{t-1}$. The ${x}_{t}$ at moment t is linearly transformed with the information splice at moment t-1, multiplied by the weight matrix ${W}_{r}$, and then passed through the activation function to obtain the output.

1 $$r_{t} = \sigma (W_{r} \cdot [h_{t - 1} ,x_{t} ])$$

The second is the update gate, which controls how much of the hidden state ${h}_{t}$ of the current moment in the data sequence is retained from the hidden state of the historical moment and how much new information is received from the candidate state at the current moment. The update gate is calculated in the same way as the reset gate, except that the parameters of the linear transformation are changed.

2 $${\text{z}}_{t} = \sigma (W_{z} \cdot [h_{t - 1} ,x_{t} ])$$

Then comes the candidate state, which is obtained by multiplying the reset gate with the hidden state ${h}_{t-1}$ at moment t-1, mainly to determine the historical information available in the previous step, followed by a linear transformation, after which the tanh activation function results in $\widetilde{{\mathrm{h}}_{\mathrm{t}}}$, indicating the current memory retention to the final memory information.

3 $${\tilde{\text{h}}}_{t} = \tanh (W_{{\tilde{h}}} \cdot [r_{t} *h_{t - 1} ,x_{t} ])$$

Finally, the GRU multiplies the value 1-${z}_{t}$ of the update gate output with the hidden state ${h}_{t-1}$ at moment t-1 based on the above-computed output to determine the final memory information retained at moment t-1. The ${z}_{t}$ of the update gate output is then stitched with the result obtained from the candidate state as the final hidden state output ${h}_{t}$, which can alleviate the problem of gradient disappearance to a certain extent.

4 $${\text{h}}_{{\text{t}}} = (1 - z_{t} )*h_{t - 1} + z_{t} *\tilde{h}_{t}$$

**Fig. 4:**
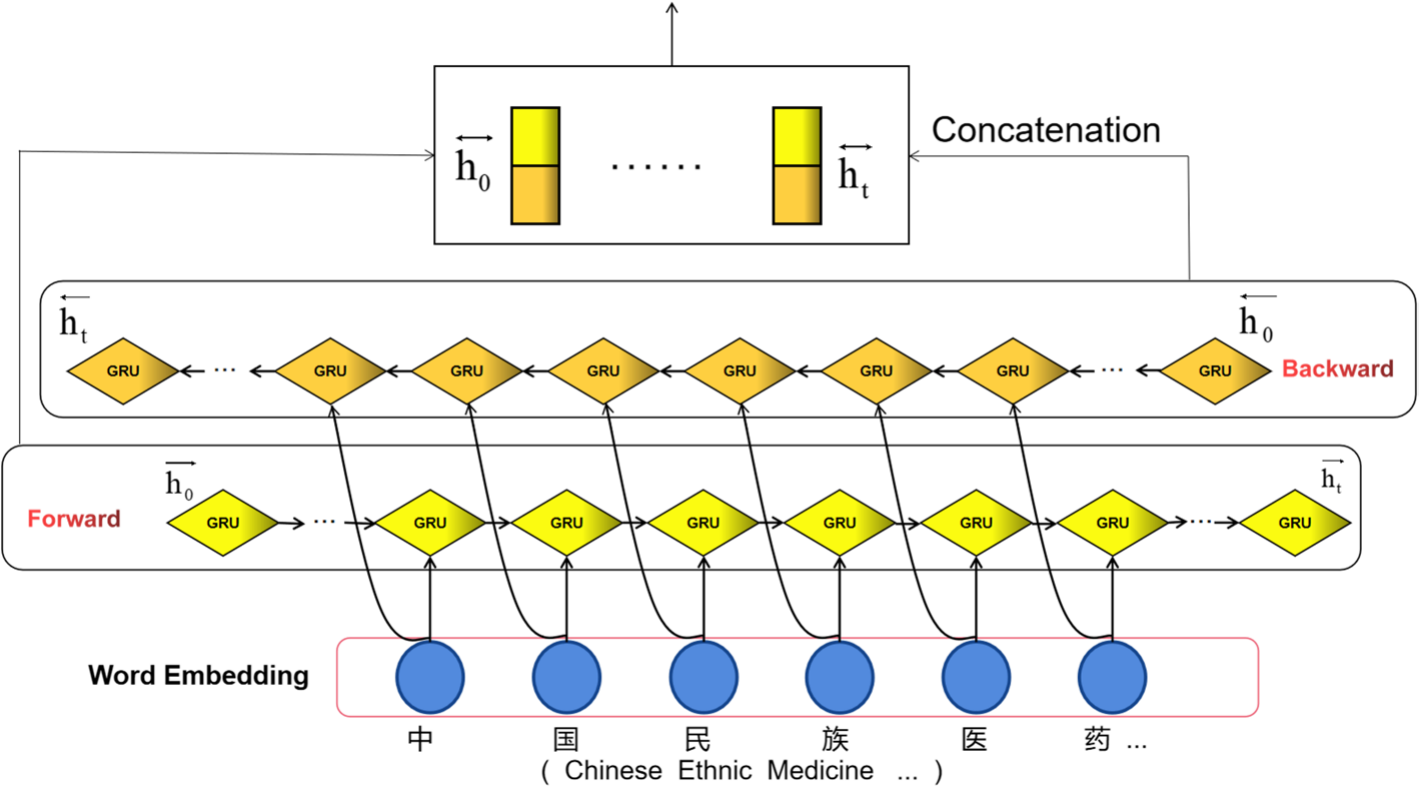
BiGRU model structure

One-way GRU is transmitted from backward to forwards, which ignores the influence of later words on the overall logical state so that with BiGRU, the contextual features of the question are captured and semantic associations are reinforced by two-way transmission. The vector matrix output from the word embedding layer is extracted to deep global semantic features by forwarding GRU and backward GRU networks. The model structure of BiGRU is as pictured in Fig. 4.

BiGRU receives the feature information of the hidden layer from the forward and backward directions. The state of the hidden layer at the current moment t is determined by the input${x}_{t}$, the forward hidden layer state $\overrightarrow{{\mathrm{h}}_{\mathrm{t}-1}}$ and the backward hidden layer state $\overleftarrow{{h}_{t-1}}$ at the moment t-1 together, which is eventually obtained by a forward and backward weighted summation. The formula is as follows.

5 $$\overrightarrow {{h_{t} }} = GRU(x_{t} ,\overrightarrow {{h_{t - 1} }} )$$

6 $$\overleftarrow {{h_{t} }} = GRU(x_{t} ,\overleftarrow {{h_{t - 1} }} )$$

7 $$h_{t} = w_{t} \overrightarrow {{h_{t} }} + v_{t} \overleftarrow {{h_{t} }} + b_{t}$$

where the GRU function is a non-linear fusion of the input word vectors, encoding the word vectors into the corresponding GRU hidden layer states;${w}_{t}$, ${v}_{t}$ are the weights corresponding to $\overrightarrow{{h}_{t}}$ and $\overleftarrow{{\mathrm{h}}_{t}}$ for the BiGRU at moment t, and ${b}_{t}$ denotes the bias corresponding to the hidden layer states.

#### Attention mechanism

**Fig. 5:**
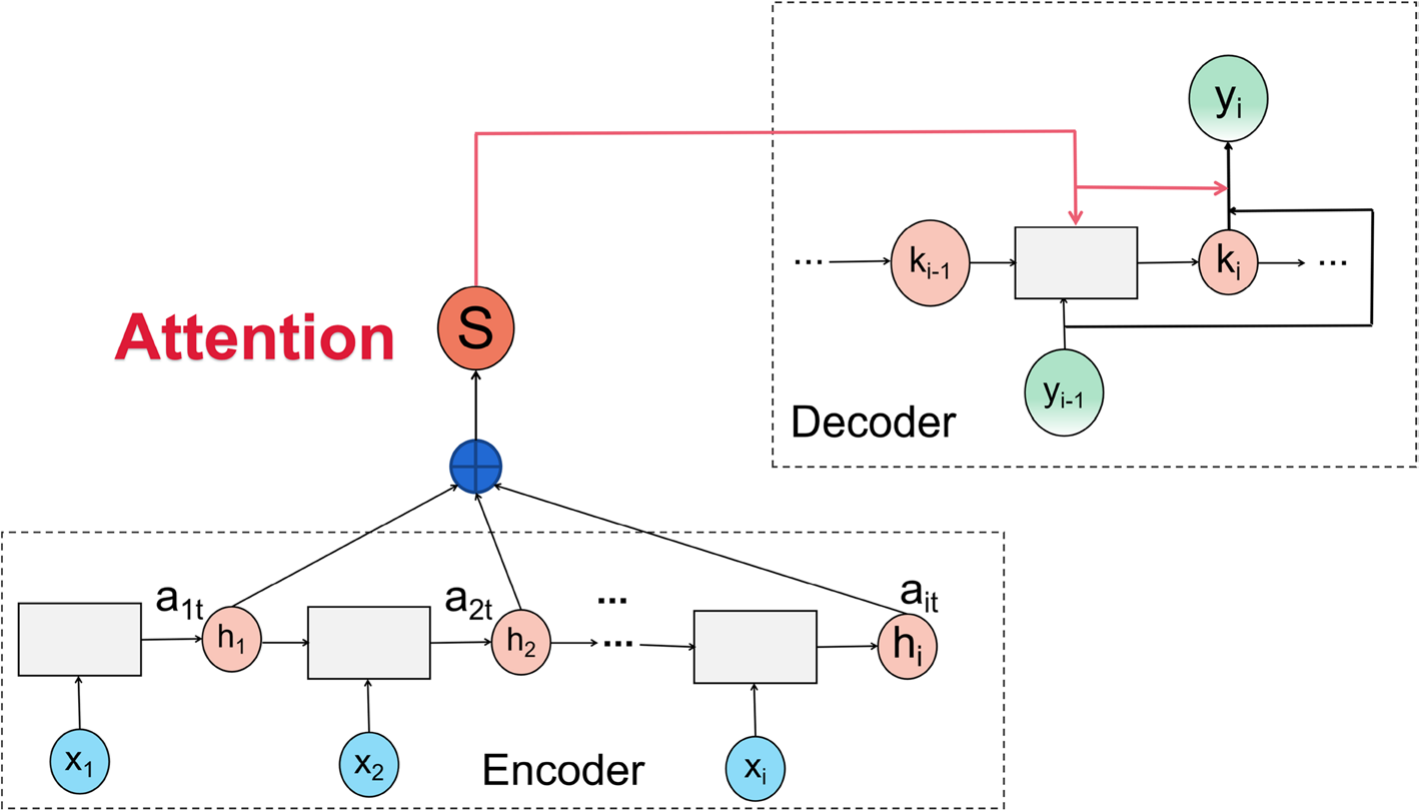
Structure of attention mechanism

The attention mechanism is introduced after BiGRU, which mainly measures the feature weights of the hidden layer vectors, calculates the output weights at different moments, allocates corresponding weights to the word vectors with different degrees of important information, and extracts keywords feature information. The attention mechanism is implemented as seen in Fig. 5.

The input to the attention mechanism layer is the feature vector h_it_ output from the previous BiGRU network layer processed by the dense layer, ${u}_{it}$ is the hidden representation of the multilayer perceptron ${h}_{it}$, ${w}_{w}$ represents the weight matrix, ${b}_{w}$ represents the bias. The formula is as follows.

8 $${\text{u}}_{it} = \tanh (w_{{\text{w}}} h_{it} + b_{w} )$$

${u}_{w}$ is a randomly initialized attention matrix, which is accumulated by multiplying the different probability weights assigned to the individual hidden layer states to obtain the normalized weights ${a}_{it}$ to the softmax function. The formula is as follows.

9 $${\text{a}}_{{{\text{it}}}} = \frac{{\exp (u_{{{\text{it}}}}^{T} u_{{\text{w}}} )}}{{\sum\limits_{t} {\exp (u_{it}^{T} u_{w} )} }}$$

The final weighted feature vector is extracted, which contains the crucial feature information of the interrogative sentence.

10 $$S = \sum\limits_{t = 1}^{n} {a_{it} h_{it} }$$

#### CNN

After the BiGRU layer gets the contextual feature information, capturing the global dependency features, the attention mechanism assigns different probability weights to its output, the larger the weight, the more semantically critical the question sentence is, which means more focused attention, and then through the convolutional neural network to get the local features of the question sentence, using multiple convolution kernels of different sizes to extract the deeper semantically critical features in the question sentence, to ensure the feature information such as information relevance and location invariance over long distances. The CNN comprises a convolutional layer and a pooling layer. The convolutional operation is performed by a filter of h*n dimensional size, where h denotes the convolution kernel size, n stands for the word vector dimension, H is the input matrix and S is the convolution kernel weight matrix. The formula is as follows.

11 $$c_{i} = f(S \bullet H_{i:i + h - 1} + b)$$

where $f$ is the Relu [39] nonlinear activation function, b is the bias term and ${H}_{i:i+h-1}$ is the feature ci extracted after the convolution operation. Depending on the above results, the final convolution calculation is performed on the feature matrix of each window, and $k$ is the number of word vectors for the question.

12 $$c = (c_{1} ,c_{2} , \cdots ,c_{k - h + 1} )$$

_The pooling layer is mainly responsible for filtering the unimportant interrogative features derived from the convolutional layer and retaining the most critical text feature information. The pooling operation is divided into a maximum pooling layer and an average pooling layer. The global maximum pooling process is adopted here, which enables the dimensionality reduction of the vector while avoiding overfitting phenomena, and the output vector is gained after the pooling process._

13 $$P = \max (c_{i} )$$

_The multiple feature vectors obtained after pooling are reintegrated and served as input for similarity calculation, effectively reducing the loss of feature information and ultimately the semantic vector of interrogative sentences is learned._

#### Output layer

The final output vectors after processing by the Siamese network model are calculated with Manhattan distance to ascertain whether they are semantically similar, that is whether the spatial distances are similar. Similar features are found by calculating the absolute distance between two points x and y on the spatial coordinate axis with the following formula.

14 $${\text{Dist}}(Manhatta{\text{n}}) = |x_{1} - x_{2} | + |y_{1} - y_{2} |$$

where $x1$ and $y1$ are the outputs of the left model in the concatenated network, $x2$ and $y2$ are the outputs of the right model, and the absolute value difference between them represents the interrogative similarity measure. The last obtained feature vector is processed by the fully concatenated layer to output the final result.

### Experimental analysis

#### Data sets

This paper focuses on the ethnic medical question datasets, which contain a total of 22,655 question pairs. A question pair consists of two questions and a label, with the label set to 1 if the two questions have the same semantic meaning and 0 if the opposite is true, with half of the question pairs having labels 0 and 1 each. During the experimental training, the datasets are divided into a training set and a test set according to 8:2, with 18,124 entries in the training set and 4531 entries in the test set. To validate the effectiveness of the model proposed in this paper, a comparison experiment with the Novel Coronavirus Pneumonia 2019 medical public datasets is also established (https://www.heywhale.com/mw/dataset/5fd1934e1a34b90030b6074d).

#### Experimental environment and parameter settings

##### Experimental environment

The specific experimental environment setup for this paper is illustrated in Table 3.

**Table 3:** Experimental environment configuration

Experimental environment	Configuration
Operating system	64-bit Windows 10
Hardware platform	NVIDIA GeForce GTX 1650
Programming environment	Pycharm
Programming language	Python 3.6
Deep learning framework	Keras 2.2.5
Central processing unit	Intel(R) Core(TM) i5-9300H CPU @ 2.40 GHz

##### Experimental parameter settings

The Word2vec tool was used to convert the datasets into a model-readable word vector, with arbitrary values in the range of 50 to 400. After several iterations of training, it was found that the best accuracy was achieved when the embedding dim was equal to 100, and the model parameters were set as listed in Table 4 below.

**Table 4:** Main parameter settings of the model

Parameter name	Parameter values
Word vector dimension	100
Epochs	40
Learning_rate	0.005
Hidden_nums	100
Kernel_size	5
optimizer	Adam
dropout	0.5
Batch_size	64

#### Evaluation indicators

In this paper, accuracy, precision, recall and F1-score evaluation metrics are adopted to measure the performance of the model. The evaluation metric parameters are given in Table 5. With TP representing actual positive samples and predicted positive samples, FN denoting actual positive samples and predicted negative samples, FP for actual negative samples but predicted positive samples, and TN in which actual negative samples are predicted to be negative samples.

**Table 5:** Evaluation index parameters

	Predicted value	
True value	Positive	Negative
True	True Positive(TP)	False Negative(FN)
False	False Positive(FP)	True Negative (TN)

Accuracy indicates the proportion of correctly predicted samples to the total sample, and the correctly predicted samples are TP and TN, the formula is calculated as follows.

15 $${\text{Accuracy}} = \frac{TP + TN}{{TP + TN + FP + FN}}$$

Precision refers to the proportion of true samples to those predicted to be positive, and those predicted to be positive include TP and FP with the following formula.

16 $${\text{Precision}} = \frac{TP}{{TP + FP}}$$

Recall denotes the proportion of positive cases in all samples that were correctly predicted, including TP and FN, and the formula is given below.

17 $$R{\text{ecall}} = \frac{TP}{{TP + FN}}$$

The value of F1 is calculated based on the recall and precision rates as indicated in the formula below.

18 $$F1 = \frac{{2*{\text{Precision}}*{\text{Recall}}}}{{{\text{Precision}} + {\text{Recall}}}}$$

## Result

Comparing the model proposed in this paper with other text similarity matching models, which are mainly ABCNN [40], ESIM [41], BIMPM [42], Siamese-BiLSTM, Siamese-BiGRU, Siamese-BiGRU-attention, where ABCNN is a similarity matching model built based on CNN and attention mechanism; ESIM is a hybrid neural model based on BiLSTM and treeLSTM; BIMPM is a bilateral multi-angle text matching model based on BiLSTM, and the experimental results were compared based on the ethnic medical question datasets, all vectorized using the Word2vec tool for questioning, as seen in Table 6, showing the accuracy, recall and F1-score of these kinds of models.

**Table 6:** Effectiveness of different models on the ethnic medicine question datasets (unit %)

Model	Precision	Recall	F1-score
ABCNN	86.55	83.68	85.09
ESIM	87.72	90.19	88.94
BIMPM	90.70	90.26	90.48
Siamese-BiLSTM	89.35	92.17	90.74
Siamese-BiGRU	91.49	93.38	92.43
Siamese-BiLSTM-attention	92.97	93.87	93.42
Siamese-BiGRU-attention	93.05	96.78	94.88
Siamese-BiLSTM-Attention-CNN	95.22	94.86	95.04
Siamese-BiGRU-Attention-CNN	**97.15**	**98.82**	**97.98**

Table 6 shows the comparison of different deep learning models. The ABCNN model has fewer structural levels and captures insufficient semantic information of the sentence, and the results of the training are the worst; the ESIM model extracts contextual order information of sentences and achieves an F1-score of 88.94%; the BIMPM model captures sentence feature information from multiple perspectives and achieves an F1-score of 90.48%. The results of the models built with Siamese networks generally outperformed the first three models, and the results of precision, recall, and F1-score were all significantly elevated, with those containing BiGRU models generally improving the F1 values by 1%-2% over BiLSTM, and introducing the Attention mechanism on the foundation of Siamese-BiGRU, the F1-score improved by 2%. It is suggested that the model containing the attention mechanism works somewhat better since the attention mechanism can assign more weight to the key information in the interrogative sentences, which highlights the important features that to some extent can enhance the extraction of key features of similar interrogative sentences. By incorporating the convolutional neural network on top of Siamese-BiGRU-Attention, the F1-score improved by 3%, indicating that the CNN maintained the position invariant property on significant feature information, and at the same time, on the basis of the contextual feature information of the sentences obtained by BiGRU, the rich semantic feature vector of the sentences was gotten more deeply, which compared with other models, the F1- score reached 97.98%.

**Fig. 6:**
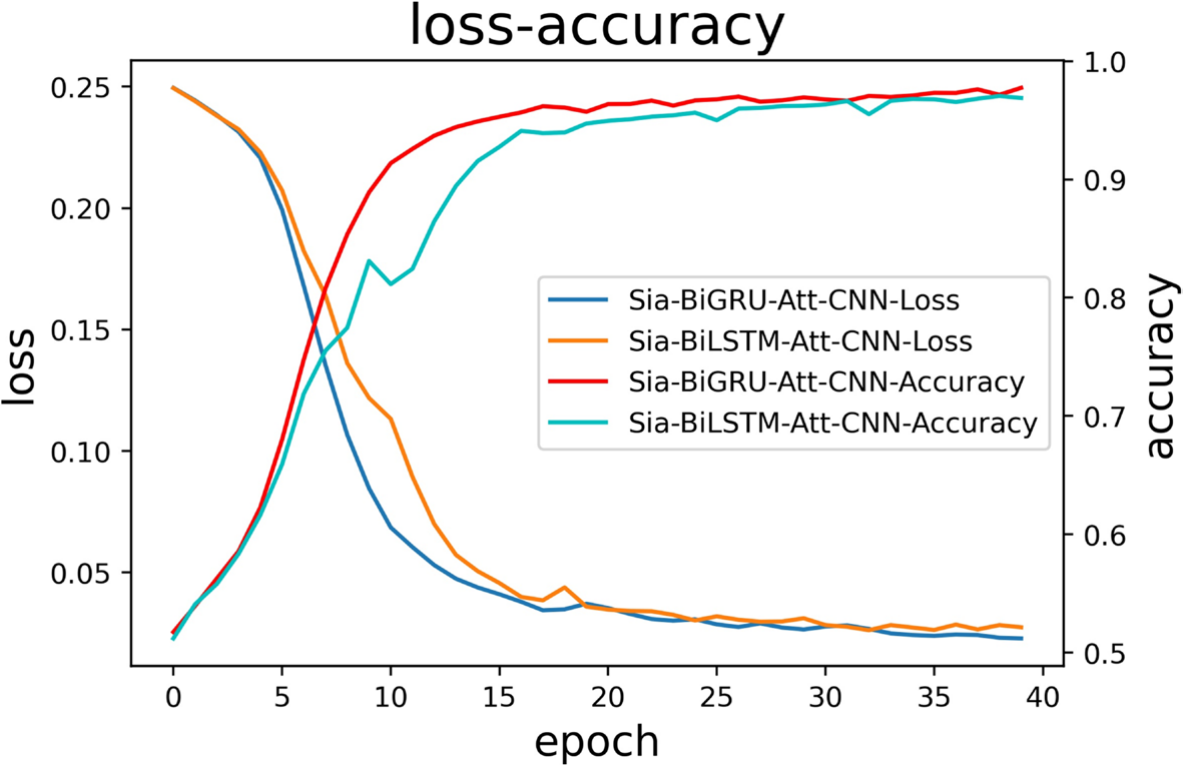
Loss-accuracy change curve

Figure 6 presents the Siamese-BiGRU-Attention-CNN compared with the Siamese-BiLSTM-Attention-CNN model, and the lower graph shows the loss and accuracy plots for both models, as displayed in Fig. 6. From the comparison of the results in the figure, it can be concluded that during the training of the model, the loss continuously decreases, and the Siamese-BiGRU-Attention-CNN converges faster, with a slightly steeper descent slope and fewer floating changes than the Siamese-BiLSTM-Attention-CNN. The accuracy gradually smoothed out at epoch 20 and reached its best at epoch 40, where the value of accuracy reached 97.24% and the value of the loss function dropped to 2.65%, with both accuracy and loss values reaching a state of convergence. During the training process, the model continuously iterated to update the parameters, making the performance continuously optimized; while the Siamese-BiLSTM-Attention-CNN exhibited a large fluctuation in accuracy at the early stage of training, and the loss value showed a decreasing trend as the number of training rounds increased, and the accuracy did not level off until the epoch was 33, with the final result being slightly lower than that of the Siamese-BiGRU-Attention-CNN. The comprehensive performance of Siamese-BiGRU-Attention-CNN is a bit better as shown by the loss-accuracy image analysis.

**Fig. 7:**
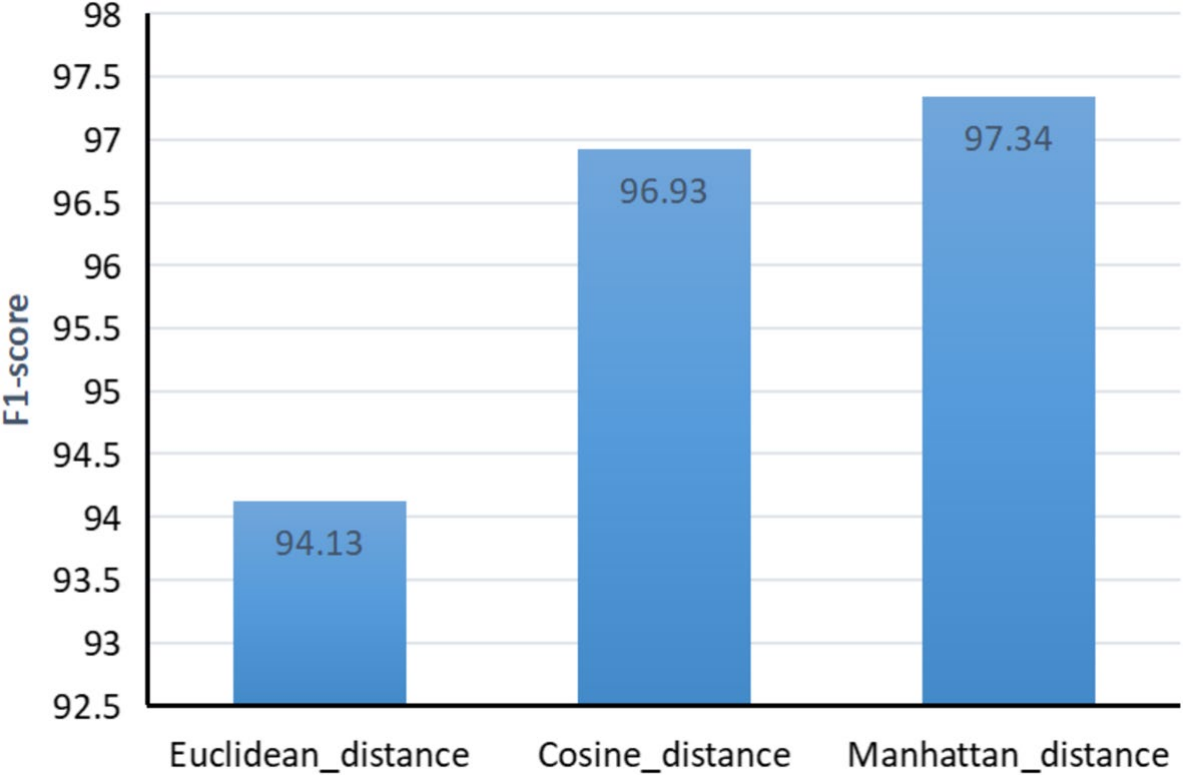
Spatial distance calculation

Based on the performance of the Siamese-BiGRU-Attention-CNN model described above, the interrogative similarity was calculated by comparing the cosine distance [43], the Euclidean distance [44] and the Manhattan distance, and as shown in Fig. 7, Manhattan works best and is the most effective metric among the similar feature vectors.

After the loss function processing, gradient optimization is needed to adjust the optimizer of the model. In this paper, three optimizers, Nadam [45], Adam [46], and RMSprop [47], were chosen to optimize the model for training, as displayed in Table 7. The Adam optimizer worked best, with faster convergence and shorter training times, and the accuracy converged more quickly with fewer iterations than the other two, so the Adam optimizer was selected for model optimization.

**Table 7:** Optimizer comparison (unit %)

Optimizer	Accuracy	Convergence Batch
Adam	97.95	40
RMSprop	97.80	59
Nadam	97.26	52

To validate the model, the Novel Coronavirus Pneumonia question set COVID 2019, the medical public datasets, were used to validate the model with the same parameter settings. Table 8 shows a sample COVID 2019 questions. The datasets have a total of 10,750 entries, the datasets sample are small and the training set and test machine are divided 8:2.

**Table 8:** Example of COVID 2019 question-sentence

Q1	Q2	label
What are the symptoms of pneumonia in a novel coronavirus infection?	What are the symptoms of coronavirus infection?	1
How is the incubation period of novel coronavirus pneumonia detected?	Can a patient with pneumonia, hypertension grade 2 and coronary arteriosclerosis have an imaging test?	0

The COVID 2019 datasets were trained on each of the following nine models, with the precision, recall, and F1-score results shown in Table 9, and similar to the results of the model runs on the ethnic medicine interrogative datasets, the ABCNN training yielded the worst results. On the Siamese-BiGRU-Attention-CNN model proposed in this paper, the precision, recall, and F1-score results of the training on the new crown question datasets outperformed other models, and the F1-score reached 97.62%, the proposed model in this paper had better performance and verified the effectiveness of the model.

**Table 9:** Effectiveness of different models on the COVID 2019 datasets (unit %)

Model	Precision	Recall	F1-score
ABCNN	88.01	79.32	83.44
ESIM	91.41	86.01	88.63
BIMPM	90.91	89.56	90.23
Siamese-BiLSTM	94.80	86.28	90.33
Siamese-BiGRU	95.53	89.17	91.06
Siamese-BiLSTM-attention	95.04	88.21	91.50
Siamese-BiGRU-attention	96.04	91.33	93.50
Siamese-BiLSTM-Attention-CNN	96.47	93.67	94.93
Siamese-BiGRU-Attention-CNN	98.16	97.19	97.62

## Discussion

We constructed datasets of ethnic medical interrogative sentences to achieve accurate matching of ethnic medical similar semantic interrogative data in this paper. Yet there is still an area for improvement in our experiments. In the first place, the model can adequately extract the important words in the interrogative sentences, but for some implicit knowledge and relationships in medicine, especially as some of the texts are still ancient medical books, which are relatively obscure for some doctors, it is more difficult for the model to learn the important associations, so the model can be further improved by consulting medical professionals to incorporate this hidden knowledge into the model and address the shortcomings. Next, by matching the similarity of the questions, the corresponding answers to the questions can be further retrieved and how to return the answers by matching the questions can be investigated.

## Conclusion

This paper is based on the Siamese network framework combined with the BiGRU model while introducing an attention mechanism and convolutional neural network for question-sentence similarity matching. Firstly, the bi-directional GRU extracts contextual order information and long-distance dependent features in the question-sentence, the attention mechanism further assigns higher weights to the key information, while the CNN can mainly capture the feature vectors with invariant local positions, so that the model can fully obtain the important feature information in the question-sentence, and eventually, the spatial distance is calculated by the Manhattan formula to receive the similarity results, which leads to a high accuracy rate. It is also possible to simulate the order of questions in different dimensions and to allocate attention to keywords in semantic sequences. The main purpose of this paper is to input ethnic medical similarity question pairs and identify whether the question pairs have similar semantics, which can be further used for medical Q&A and patient self-diagnosis online. The experimental results show that the model in this paper has a better sequence modelling capability, can reasonably assign attention weights and can utilize key semantic information to identify the similarity of ethnic medicine interrogatives. Additionally, Siamese network is not only used for similarity matching of interrogative sentences, but also has other general applications, such as local matching of images, face comparison verification, fingerprint comparison, signature verification, assessment of disease severity based on clinical grading, and other fields. By taking advantage of the Siamese network with two identical structures and shared weights, more useful feature information can be captured to help evaluate the similarity of the input samples.

Based on this research approach of this paper, it can match the questions asked by patients more precisely and have a deeper semantic understanding of their needs, which helps save resources and time, and facilitates communication between doctors and patients. It also helps to save resources and time, and facilitates the communication between doctors and patients. It raises the level of medical technology and medical quality of doctors, and meets the needs of people who use ethnic medicine to protect their health.

## Data Availability

The datasets used and analyzed during the current study are available from the corresponding author on reasonable request.

## References

[1] Alqifari R (2019). Question answering systems approaches and challenges. Proc Stud Res Workshop Assoc RANLP.

[2] Slater LT, Karwath A, Williams JA (2021). Towards similarity-based differential diagnostics for common diseases. Comput Biol Med.

[3] Harispe Sébastien (2015). Semantic similarity from natural language and ontology analysis. Synth Lect Hum Lang Technol.

[4] Lu W, Huang H, Zhu C (2012). Feature words selection for knowledge-based word sense disambiguation with syntactic parsing. Przeglad Elektrotechniczny.

[5] Aliguliyev RM (2009). A new sentence similarity measure and sentence based extractive technique for automatic text summarization. Expert Syst Appl.

[6] Thangaraj M, Sivakami M (2018). Text classification techniques: a literature review. Interdiscip J Inf Knowl Manag.

[7] Chiong R, Budhi GS, Dhakal S (2021). A textual-based featuring approach for depression detection using machine learning classifiers and social media texts. Comput Biol Med.

[8] Amir S, Tanasescu A, Zighed DA (2017). Sentence similarity based on semantic kernels for intelligent text retrieval. J Intell Inf Syst.

[9] Sarrouti M, El Alaoui SO (2020). SemBioNLQA: A semantic biomedical question answering system for retrieving exact and ideal answers to natural language questions. Artif Intell Med.

[10] Yih SW, Chang MW, Meek C (2013). Question answering using enhanced lexical semantic models. Proceedings of the 51st Annual Meeting of the Association for Computational Linguistics.

[11] Bär D, Biemann C, Gurevych I, et al. Ukp: Computing semantic textual similarity by combining multiple content similarity measures* SEM 2012: The First Joint Conference on Lexical and Computational Semantics–Volume 1: Proceedings of the main conference and the shared task, and Volume 2: Proceedings of the Sixth International Workshop on Semantic Evaluation (SemEval 2012). 2012. p. 435–40.

[12] Jimenez S, Becerra C, Gelbukh A. Soft cardinality: A parameterized similarity function for text comparison* SEM 2012: The First Joint Conference on Lexical and Computational Semantics–Volume 1: Proceedings of the main conference and the shared task, and Volume 2: Proceedings of the Sixth International Workshop on Semantic Evaluation (SemEval 2012). 2012. p. 449–53.

[13] Qaiser S, Ali R (2018). Text mining: use of TF-IDF to examine the relevance of words to documents. Int J Comput Appl.

[14] Kondrak G (2005). N-gram similarity and distance[C]//International symposium on string processing and information retrieval.

[15] Sadowski C, Levin G (2007). Simhash: Hash-based similarity detection.

[16] Niwattanakul S, Singthongchai J, Naenudorn E (2013). Using of Jaccard coefficient for keywords similarity. Proc Int Multiconf Eng Comput Sci..

[17] He H, Gimpel K, Lin J (2015). Multi-perspective sentence similarity modeling with convolutional neural networks. Proceedings of the 2015 conference on empirical methods in natural language processing.

[18] Shi-ying F, Wen-tin H (2018). Accelerating recurrent neural network training based on speech recognition model. J Chin Comput Syst.

[19] Hochreiter S, Schmidhuber J (1997). Long short-term memory. Neural Comput.

[20] Huang PS, He X, Gao J (2013). Learning deep structured semantic models for web search using clickthrough data. Proceedings of the 22nd ACM international conference on Information & Knowledge Management.

[21] Bromley J, Bentz J, Bottou L, Guyon I, Lecun Y, Moore C, Sackinger E, Shah R. Signature Verification using a "Siamese" Time Delay Neural Network[J]. International Journal of Pattern Recognition and Artificial Intelligence. 1993;7:25.

[22] Shen Y, He X, Gao J (2014). A latent semantic model with convolutional-pooling structure for information retrieval. Proceedings of the 23rd ACM international conference on conference on information and knowledge management.

[23] Hu B T, Lu Z D, Li H, Chen Q C. Convolutional Neural Network Architectures for Matching Natural Language Sentences[C]. 28th Conference on Neural Information Processing Systems (NIPS). 2014:2042–50.

[24] Palangi H, Deng L, Shen Y, et al. Semantic modelling with long-short-term memory for information retrieval. arXiv preprint arXiv:1412.6629, 2014.

[25] Mueller J, Thyagarajan A, Aaai. Siamese Recurrent Architectures for Learning Sentence Similarity[C]. 30th Association-for-the-Advancement-of-Artificial-Intelligence (AAAI) Conference on Artificial Intelligence. 2016:2786–92.

[26] Neysiani B S, Babamir S M, IEEE. New Methodology for Contextual Features Usage in Duplicate Bug Reports Detection[C]. 5th International Conference on Web Research (ICWR). 2019:178–83.

[27] Neculoiu P, Versteegh M, Rotaru M (2016). Learning text similarity with siamese recurrent networks. Proceedings of the 1st Workshop on Representation Learning for NLP.

[28] Chung J, Gulcehre C, Cho K H, et al. Empirical evaluation of gated recurrent neural networks on sequence modeling. arXiv preprint arXiv:1412.3555, 2014.

[29] Srivastava Nitish (2014). Dropout: a simple way to prevent neural networks from overfitting. J Mach Learn Res.

[30] Semeniuta S, Barth E. Image Classification with Recurrent Attention Models[C]. IEEE Symposium Series on Computational Intelligence (IEEE SSCI). 2016:1–7.

[31] Bertinetto L, Valmadre J, Henriques JF (2016). Fully-convolutional siamese networks for object tracking. European conference on computer vision.

[32] Che W, Li Z, Liu T (2010). Ltp: A chinese language technology platform. Coling 2010: Demonstrations.

[33] Junyi S. jieba. https://github.com/fxsjy/jiebaReturn to ref 25 in article https://github.com/fxsjy/jieba

[34] Levy O, Goldberg Y. Neural Word Embedding as Implicit Matrix Factorization[C]. 28th Conference on Neural Information Processing Systems (NIPS). 2014.

[35] Sarzynska-Wawer J, Wawer A, Pawlak A, Szymanowska J, Stefaniak I, Jarkiewicz M, Okruszek L. Detecting formal thought disorder by deep contextualized word representations[J]. Psychiatry Research. 2021;304:114135. 10.1016/j.psychres.2021.114135 34343877

[36] Pennington J, Socher R, Manning CD (2014). Glove: Global vectors for word representation. Proceedings of the 2014 conference on empirical methods in natural language processing (EMNLP).

[37] Devlin J, Chang M W, Lee K, et al. Bert: Pre-training of deep bidirectional transformers for language understanding. arXiv preprint arXiv:1810.04805, 2018.

[38] Mikolov T, Sutskever I, Chen K, Corrado G, Dean J. Distributed representations ofwords and phrases and their compositionality[C]. 27th Annual Conference on Neural Information Processing Systems, (NIPS). 2013.

[39] Glorot X, Bordes A, Bengio Y. Deep sparse rectifier neural networks[C]. Proceedings of the fourteenth international conference on artificial intelligence and statistics. 2011:315–23.

[40] Yin W, Schütze H, Xiang B (2016). Abcnn: Attention-based convolutional neural network for modeling sentence pairs. Trans Assoc Comput Linguist.

[41] Chen Q, Zhu X, Ling Z, et al. Enhanced LSTM for natural language inference. arXiv preprint arXiv:1609.06038, 2016.

[42] Graves A, Schmidhuber J (2005). Framewise phoneme classification with bidirectional LSTM and other neural network architectures. Neural Netw.

[43] Liao H, Xu Z (2015). Approaches to manage hesitant fuzzy linguistic information based on the cosine distance and similarity measures for HFLTSs and their application in qualitative decision making. Expert Syst Appl.

[44] Elmore KL, Richman MB (2001). Euclidean distance as a similarity metric for principal component analysis. Mon Weather Rev.

[45] Wang J, Cao Z W. Chinese Text Sentiment Analysis Using LSTM Network Based on L2 and Nadam[C]. IEEE 17th International Conference on Communication Technology (ICCT). 2017:1891–95.

[46] Zhang Z. Improved adam optimizer for deep neural networks[C]. 2018 IEEE/ACM 26th international symposium on quality of service (IWQoS). 2018:1-2.

[47] Babu DV, Karthikeyan C, Kumar A (2020). Performance analysis of cost and accuracy for whale swarm and rmsprop optimizer[C]//IOP Conference Series: Materials Science and Engineering. IOP Publishing.

